# Supplementary material for: Integrated control of transporter endocytosis and recycling by the arrestin-related protein Rod1 and the ubiquitin ligase Rsp5
Source: eLife. 2014 Nov 7;3:e03307. doi: 10.7554/eLife.03307 (PMC4244573; doi:10.7554/eLife.03307)
Supplement: Supplementary file 1. — A table listing yeast strains used in this study is provided in Supplementary file 1. DOI: http://dx.doi.org/10.7554/eLife.03307.033 [file elife03307s001.docx]

**SUPPLEMENTARY FILE**

**Supplementary file 1.** Yeast strains used in this study.

| **Strain (Background),**  **Description** | **Genotype** | **Origin / Reference** |
| --- | --- | --- |
| **ySL542** (BY4741)  WT, Rod1-GFP | Mat a ; *his3∆1 leu2∆0 met15∆0 ura3∆0*  *ROD1:*GFP-*HisMX3* | This study |
| **ySL600** (BY4741)  *reg1∆*, Rod1-GFP | Mat a ; *his3∆1 leu2∆0 met15∆0 ura3∆0*  *reg1∆::KanMX ; ROD1:*GFP-*HisMX3* | This study |
| **ySL638** (BY4741)  WT, Sec7-mCh, Rod1-GFP | Mat a ; *his3∆1 leu2∆0 met15∆0 ura3∆0*  *SEC7:*mCh*-KanMX ;* pSL93 (p416-*p_ROD1_*-*ROD1*-GFP) | This study |
| **ySL743** (BY4741)  *rod1*∆, Jen1-GFP | Mat a ; *his3∆1 leu2∆0 met15∆0 ura3∆0*  *rod1∆::Hph-NT1* *JEN1*:GFP-*HisMX3* | This study |
| **ySL781** (BY4741)  *rod1*∆, p*_GAL_*:Jen1-GFP | Mat a ; *his3∆1 leu2∆0 met15∆0 ura3∆0*  *rod1*∆::*HisMx* ; pRHT373 (p416, *p_GAL_*:*JEN1*-GFP-HIS6) | Becuwe et al., 2012b |
| **ySL956** (BY4741)  WT, Dip5-GFP, Sec7-mCh | Mat a ; *his3∆1 leu2∆0 met15∆0 ura3∆0*  *DIP5-*GFP*-HisMX ; SEC7*:mCh-*NatNT2* | This study |
| **ySL1011** (BY4741)  WT, Sec7-mCh, GFP-Rsp5 | Mat a ; *his3∆1 leu2∆0 met15∆0 ura3∆0*  *SEC7:*mCh*-KanMX ;* pSL29 *(*p416- *p_CYC_*-GFP-Rsp5) | This study |
| **ySL1083** (BY4741)  WT, p*_GAL_*:Jen1-GFP | Mat a ; *his3∆1 leu2∆0 met15∆0 ura3∆0*  pRHT373 (p416, *p_GAL_*:*JEN1*-GFP-HIS6) | Becuwe et al., 2012b |
| **ySL1146** (BY4741)  WT, Stl1-GFP | Mat a ; *his3∆1 leu2∆0 met15∆0 ura3∆0*  *STL1:*GFP*-HISMX* | This study |
| **ySL1150** (BY4741)  WT, Jen1-GFP | Mat a ; *his3∆1 leu2∆0 met15∆0 ura3∆0*  *Jen1*-GFP:*Hph-NT1* | This study |
| **ySL1153** (BY4741)  *rod1∆*, Stl1-GFP | Mat a ; *his3∆1 leu2∆0 met15∆0 ura3∆0*  *rod1*∆*::KanMX ; STL1:*GFP*-HISMX* | This study |
| **ySL1165** (BY4741)  WT, Jen1-GFP, Sec7-mCh | Mat a ; *his3∆1 leu2∆0 met15∆0 ura3∆0*  *Jen1*-GFP:*Hph-NT1 : SEC7-*mCh:*KanMX* | This study |
| **ySL1168** (BY4741)  WT, Jen1-GFP, Vps17mCh | Mat a ; *his3∆1 leu2∆0 met15∆0 ura3∆0*  *Jen1*-GFP:*Hph-NT1 : VPS17-*mCh:*KanMX* | This study |
| **ySL1175** (BY4741)  *ypt6*∆, Jen1-GFP | Mat a ; *his3∆1 leu2∆0 met15∆0 ura3∆0* *ypt6∆::KanMX ; JEN1*:GFP-*Hph*-*NT1* | This study |
| **ySL1176** (BY4741)  *rod1*∆, Jen1-GFP, Sec7-mCh | Mat a ; *his3∆1 leu2∆0 met15∆0 ura3∆0*  *rod1∆::HisMx ; Jen1*:GFP-*HphNT1 ; SEC7:*mCh-*KanMX* | This study |
| **ySL1177** (BY4741)  *rod1*∆, Jen1-GFP, Vps17-mCh | Mat a ; *his3∆1 leu2∆0 met15∆0 ura3∆0*  *rod1∆::HisMx ; Jen1*:GFP-*HphNT1 ; VPS17:*mCh-*KanMX* | This study |
| **ySL1307** (yAB852)  *gga1*∆ *gga2*∆, Jen1-GFP, Sec7-mCh | Mat a ; *leu2 ura3 ; gga1∆::TRP1 ; gga2∆::HIS3 ; JEN1*:GFP-*Hph*-*NT1 ; SEC7:*mCh*-KanMX* | This study  (yAB852 from Scott et al., 2004) |
| **ySL1308** (yAB852)  *gga1*∆ *gga2*∆, Gga2-HA, Jen1-GFP | Mat a *; leu2 ura3 ; gga1∆::TRP1 ; gga2∆::HIS3 ; GGA2-HA:ADE3:LEU2 ; JEN1*:GFP-*Hph*-*NT1* | This study  (yAB852 from Scott et al., 2004) |
| **ySL1310** (yAB852)  *gga1*∆ *gga2*∆, p*_GAL_*:Jen1-GFP, Sec7-mCh | Mat a *; leu2 ura3 ; gga1∆::TRP1 ; gga2∆::HIS3 ;* pRHT373 (p416, *p_GAL_*:*JEN1*-GFP-HIS6) | This study  (yAB852 from Scott et al., 2004) |
| **ySL1311** (yAB852)  *gga1*∆ *gga2*∆, Gga2-HA, p*_GAL_*:Jen1-GFP | Mat a *; leu2 ura3 ; gga1∆::TRP1 ; gga2∆::HIS3 ; GGA2-HA:LEU2 ;* pRHT373 (p416, *p_GAL_*:*JEN1*-GFP-HIS6) | This study  (yAB852 from Scott et al., 2004) |
| **ySL1318** (EN60)  *9-arrestin*, Jen1-GFP | *ecm21∆::KanMX csr2∆::KanMX bsd2 rog3∆::natMX rod1∆ ygr068c∆ aly2∆ aly1∆ ldb19∆ ylr392c∆::HIS*  *his3 ura3 leu2 ; JEN1:*GFP-*Hph-NT1* | This study  (EN60 From Nikko et al., 2009) |
| **ySL1322** (yAB852)  *gga1*∆ *gga2*∆, Gga2-HA, Dip5-GFP | Mat a ; *leu2 ura3 ; gga1∆::TRP1 ; gga2∆::HIS3 ; GGA2-HA:LEU2 ; DIP5:*GFP*-Hph-NT1* | This study  (yAB852 from Scott et al., 2004) |
| **ySL1323** (yAB852)  *gga1*∆ *gga2*∆, Dip5-GFP, Sec7-mCh | Mat a ; *leu2 ura3 ; gga1∆::TRP1 ; gga2∆::HIS3 ; DIP5:*GFP*-Hph-NT1 ; SEC7*:mCh-*NatNT2* | This study  (yAB852 from Scott et al., 2004) |
| **ySL1339** (BY4741)  WT, p*_GAL_*:Jen1-KR-GFP-His6 | Mat a ; *his3∆1 leu2∆0 met15∆0 ura3∆0*  pSL184 (p416, *p_GAL_*:JEN1-KR-GFP) | This study |
| **ySL1369** (BY4741)  *vps52*∆, Jen1-GFP | Mat a ; *his3∆1 leu2∆0 met15∆0 ura3∆0* *vps52∆::KanMX ; JEN1*:GFP-*Hph*-*NT1* | This study |
| **ySL1526** (BY4741)  *ypt6∆*, Jen1-GFP, Sec7-mCh | Mat α; *his3∆1 leu2∆0 lys2∆0 ura3∆0*  *ypt6*∆::*HisMx3* *SEC7*:mCh-KanMX, *JEN1*:GFP-*Hph*-*NT1* | This study |
| **ySL1531** (BY4741)  Sec7-GFP, Vps17-mCh | Mat a ; *his3∆1 leu2∆0 met15∆0 ura3∆0*  *SEC7*:GFP-HisMX3, *Vps17*:mCh-*KanMX* | This study |
| **ySL1556** (BY4741)  *npi1*, p*_GAL_*:Jen1-GFP, | Mat a ; *his3∆1 leu2∆0 met15∆0 ura3∆0* *; promYER125W::KanMX ;* pRHT373 (p416, *p_GAL_*:*JEN1*-GFP-HIS6) | This study |
| **ySL1602** (BY4741)  *rod1∆*, Sec7-GFP, Vps17-mCh | Mat a ; *his3∆1 leu2∆0 met15∆0 ura3∆0*, *art4∆::HphNT1, SEC7-GFP::HisMX ; VPS17-mCh::KanMX ;* | This study |
| **ySL1610** (BY4741)  *vrp1∆*, Jen1-GFP | Mat a ; *his3∆1 leu2∆0 met15∆0 ura3∆0*  *vrp1∆::KanMX ;* pRHT373 (p416, *p_GAL_*:*JEN1*-GFP-HIS6) | This study |
| **ySL1615** (yAB852)  *gga1∆ gga2∆*, Vps17-GFP, Sec7-mCh | Mat a ; *leu2 ura3 ; gga1∆::TRP1 ; gga2∆::HIS3 ; VPS17-GFP::HphNT2 ; SEC7-GFP::NatNT1* | This study  (yAB852 from Scott et al., 2004) |
| **ySL1619** (yAB852)  *gga1∆ gga2∆*, Jen1-GFP, Sec7-mCh | Mat a ; *leu2 ura3 ; gga1∆::TRP1 ; gga2∆::HIS3 ; GGA2-HA:LEU2 ; JEN1*:GFP-*Hph*-*NT1 ; SEC7-mCh::KanMX* | This study  (yAB852 from Scott et al., 2004) |
| **ySL1622** (BY4741)  Jen1-GFP, Sec7-mCh, BFP-Rsp5 | Mat a ; *his3∆1 leu2∆0 met15∆0 ura3∆0*, *SEC7*:mCh-KanMX, *JEN1*:GFP-*Hph*-*NT1,* pSL303 (p415, *p_ADH_*:BFP-Rsp5) | This study |
| **ySL1625** (BY4741)  Rod1-GFP, Sec7-mCh, BFP-Rsp5 | Mat a ; *his3∆1 leu2∆0 met15∆0 ura3∆0*, *SEC7*:mCh-KanMX, pSL93 (p416-*p_ROD1_*-*ROD1*-GFP)*,* pSL303 (p415, *p_ADH_*:BFP-Rsp5) | This study |
| **ySL1630** (BY4741)  *ric1∆*, Jen1-GFP | Mat a ; *his3∆1 leu2∆0 met15∆0 ura3∆0*  *ric1∆::KanMX ; JEN1*:GFP-*Hph*-*NT1* | This study |
| **ySL1631** (BY4741)  *rgp1∆*, Jen1-GFP | Mat a ; *his3∆1 leu2∆0 met15∆0 ura3∆0*  *rgp1∆::KanMX ; JEN1*:GFP-*Hph*-*NT1* | This study |
| **ySL1636** (23344c)  WT, Jen1-GFP | *ura3, JEN1*:GFP-*Hph*-*NT1* | This study  (23 344c from Lauwers et al., 2009) |
| **ySL1638** (23344c)  *gga1∆ gga2*∆, Jen1-GFP | *ura3, gga1∆ gga2*∆, *JEN1*:GFP-*Hph*-*NT1* | This study  (*gga1*∆ *gga2*∆ from Lauwers et al., 2009: JA445) |
| **ySL1639** (23344c)  *gga1∆ gga2*∆ *ypt6*∆, Jen1-GFP | *ura3, gga1∆ gga2*∆ *ypt6*∆, *JEN1*:GFP-*Hph*-*NT1* | This study  (*gga1*∆ *gga2*∆ *ypt6*∆ from Lauwers et al., 2009: CJ036) |
| **ySL1650**(BY4741)  *vrp1*∆*,* p*_GAL_*:Jen1-GFP | Mat a ; *his3∆1 leu2∆0 met15∆0 ura3∆0*  *vrp1∆::KanMX ;* pRHT373 (p416, *p_GAL_*:*JEN1*-GFP-HIS6) | This study |
